# Prevalence of Rape and Its Predictors among Female Students Attending Elementary Schools: In the Case of Kule Refugee Camp, Gambella, Southwest Ethiopia—A Cross-Sectional Study

**DOI:** 10.1155/2023/5559246

**Published:** 2023-06-02

**Authors:** Bhan Sudan Kong, Wubishet Gezimu, Ababo Demeke, Abdissa Duguma

**Affiliations:** ^1^Department of Nursing, College of Health Sciences, Mettu University, Mettu, Ethiopia; ^2^Department of Nursing, College of Health Sciences, Dilla University, Dilla, Ethiopia

## Abstract

**Background:**

Tragically, rape victims keep their ailments a secret from the police and their family members or significant others out of concern for societal stigma. The prevalence and severity of rape are highest among minorities, including girls and children who live as refugees. The current study assessed the prevalence of rape and its predictors among female students attending elementary schools in the Kule refugee camp, Gambella, southwest Ethiopia.

**Methods:**

An institution-based cross-sectional study was conducted from May 15 to 25, 2022, using an interviewer-administered structured questionnaire. A total of 211 participants were selected using a simple random sampling technique. The collected data were entered into EpiData and then exported to SPSS version 23 for analysis. The descriptive statistics were presented using frequencies, means, and standard deviations. A binary logistic regression model was used to test the association between the outcome and explanatory variables. The multivariable analysis included variables with *p* values of less than 0.25. Finally, statistical significance was declared at a *p* value of less than 0.05.

**Results:**

A total of 210 participants were involved in this study, which has a 99.5% response rate. Of these, 73 (34.8%) were subjected to rape. Shockingly, the majority (79.5%) of those who experienced rape reported that their perpetrator did not use a condom. Smoking (AOR: 4.3; 95% CI: 1.61, 10.93), drinking alcohol (AOR: 3.2; 95% CI: 1.43, 7.03), and having a boyfriend (AOR: 2.81; 95% CI: 21, 4.05) were found to be factors associated with rape.

**Conclusion:**

This study found a high prevalence of rape in the study area. The study also identified that participants' behaviors, such as having a boyfriend, smoking, and drinking alcohol, predispose them to rape. Therefore, we recommend that the camp's administrative bodies and humanitarian service organizations strengthen the preventive measures against rape crime, including the reinforcement of solid laws against perpetrators.

## 1. Introduction

Rape is a form of sexual violence that is defined as the violent insertion of any part of the perpetrator's body into the victim's genitalia, anus, or mouth without the victim's consent [1]. Both men and women can be victims of rape at some point in their lives. For women, especially young children, it occurs more frequently and with more severe consequences. In Nigeria, for example, females aged 2 to 18 years made up nearly 89% of rape victims [2, 3]. Similarly, the National Violence Against Women Survey (NVAWS) revealed that more than half of female victims and almost three-fourths of male victims were raped before age 18 [4].

It has ample adverse effects on the health of the victims, including severe physical, psychological, and emotional consequences [5]. Rape has devastating effects on a woman's reproductive health, including the spread of sexually transmitted diseases (STDs), unintended pregnancies, unsafe abortions, and obstetric fistulas [5–8]. Regarding its impact on the mental status of the victim, it is more related to mental problems such as posttraumatic stress disorder (PTSD), suicide, and depression [8–11]. A major depressive episode is three times more likely to occur in rape victims. Also, suicide rates among rape victims were four times higher than those of nonraped individuals [12]. Besides its impact on the victim, rape also damages the matrimonial connections between spouses.

At the societal level, it also causes fear in schools, workplaces, neighborhoods, campuses, and cultural or religious communities [13–15]. Adolescents who were victimized by rape in school may develop a school phobia, which may cause them to drop out of school due to depression, fear, stigma, and shame. In particular, a rape perpetrated by a teacher and/or schoolmate may cause a lack of trust in the school environment. As a result, academic achievement in later life is significantly impacted by rape [3]. A study conducted by Jordan et al. revealed that students with previous rape and sexual assault histories have poorer grades [16].

Poor reporting and low legal access to rape cases are indicators of the pervasive culture of impunity for rape [17]. In America, only 16% of all rapes are reported to the police [12]. Victims of rape frequently endure stigma because of the possibility of contracting HIV, especially in communities with multicultural, religious, and traditional beliefs, such as those in Africa, where they uphold taboos and customs of shame related to rape. Similarly, victims sometimes hesitate to disclose to the police or family members what happened due to the perpetrator's intimidation. As a result, rape cases have not received adequate attention and have had an unseen impact on society, particularly in refugee camps where minors dwell [18].

The risk factors for rape are engrained in a deprived economy, political instability, prejudiced sociocultural beliefs, and individual risky behaviors. Individually risky predisposing behaviors include loss of security, dependence, substance use or abuse, poor knowledge of rights, and mental and physical incapability [19, 20].

Rape and other forms of sexual violence are among the violations of human rights that exist in all refugee camps [21, 22]. Evidence shows that rape, sexual assault, gang rape, discrimination, and stigmatization are common types of violence among refugees [23–26]. Moreover, almost entirely female refugees are the targets of rape [27].

The global prevalence of rape among refugees ranges from 0% to 90.9% [2]. In Africa, rapes have been persistently reported among the Democratic Republic of the Congo (DRC) refugees [28]. Amnesty International reported that rape was a major problem for Chad's refugee populations in 2019 [29].

Ethiopia, the third-highest refugee shelter in Africa, following Sudan and Uganda, is currently hosting around 844,589 refugees and asylum seekers, originating from South Sudan, Yemen, Sudan, Eritrea, Somalia, and other African countries. At the same time, the country is a state party to many international and regional human rights commissions, such as the United Nations High Commissioner for Refugees (UNHCR), which has been endeavoring to mitigate gender-based violence [30].

Regardless of the efforts of numerous humanitarian organizations, refugee women still face ongoing rape in the camps because of their fragility [21].

As far as we knew, very little was known about rape occurrence and its predictors among Ethiopia's refugee populations, particularly in the study area. Hence, the current study is aimed at assessing the prevalence of rape and its predictors among elementary schools in the Kule refugee camp, Gambella, southwest Ethiopia.

## 2. Methods and Materials

### 2.1. Study Period, Design, and Setting

From May 15 to 25, 2022, an institution-based cross-sectional study was conducted in the elementary schools of the Kule refugee camp. Kule refugee camp is located in Gambella Peoples' Regional State, southwest of Ethiopia. The camp was established in 2014 as a temporary home for South Sudanese refugees. Later, in 2016, it was stabilized with only 606 new refugees. Currently, it is providing humanitarian services to a total of 44,293 registered refugees. Of these, 63% and 55% are children and women, respectively. In the camp, preschools are open and run by Plan International, while permanent primary and early childhood schools are available and run by the Administration for Refugee and Returnee Affairs (ARRA). The huge majority of the refugees are farmers and pastoralists, and they profoundly depend on monthly aid [31].

### 2.2. Population and Eligibility Criteria

All female students who were attending elementary schools in the Kule refugee camp were considered the source population. All selected participants were considered the study population for this study. However, students who were unable to respond to questions or who were absent during data collection were excluded from this study.

### 2.3. Sample Size Determination

The sample size was determined using a single population proportion formula, *n* = (*Zα*/2)^2^ (1 − *P*)*P*/0.05^2^  assuming a population proportion of 15.5% [22], a 95% confidence level, and a 5% margin of error. Then, the calculated sample size was *n* = 201. Finally, considering a 5% nonresponse rate, the maximum sample size of *n* = 211 was used for this study.

### 2.4. Sampling Techniques

Of the three elementary schools in Kule camp, two (Plan International Elementary School and World Vision Elementary School) were randomly selected using a lottery method. Similarly, the study participants in each of the selected schools were selected using a lottery method (the students' roster was used as a sampling framework).

### 2.5. Study Outcome and Variables

The prevalence of rape was the outcome of this study. The sociodemographic characteristics (age, marital status, religion, ethnicity, residence camp, and grade level), individual behaviors (substance use), and family history (family income, maternal educational status, paternal educational status, family living together, receiving enough from family, and needing support from family members) were explanatory variables included in the study.

### 2.6. Operational Definitions

*Rape* refers to the violent insertion of any part of the perpetrator's body into the victim's genitalia, anus, or mouth without the victim's agreement. It was measured by a yes-or-no question.

*Refugee camps* refer to reception centers or places of detention for asylum-seekers or internally exiled people [32].

*Victim* refers to an individual who has suffered rape.

*Perpetrator* refers to a person that directly imposed raped.

### 2.7. Data Collection Tools, Quality Control, and Procedures

An interviewer-administered structured questionnaire that was adapted from the World Health Organization's (WHO) multicountry study on women's health and domestic violence was used with some modifications [33] (S1 Table). The interviews were conducted in a private room for each participant and guided by three data collectors (diploma nurses) and a supervisor. Since the study population is multilingual and uses English as a medium of instruction, an English version of the questionnaire was used.

A pretest was conducted among 5% of the sampled population at one of the nonselected schools in the study area. Then, necessary corrections were made to the study tool. The training was given to data collectors and supervisors two days before the data collection began. In addition, after the completion of the data collection, a careful check was done for data completeness.

### 2.8. Statistical Analysis

The collected data were entered into EpiData version 3.1 and then exported to SPSS version 23 for analysis. Descriptive statistics were presented using frequencies, means, and standard deviations. A binary logistic regression model was used to test the association between rape and explanatory variables. A multicollinearity test was carried out to see the correlation between independent variables by using standard error and colinearity statistics (variance inflation factor, VIF). The variables with a *p* value of less than 0.25 in the bivariate analysis were included in the multivariate analysis. The Hosmer-Lemeshow goodness-of-fit test was used to choose the best model. Finally, statistical significance was declared at a *p* value of less than 0.05.

## 3. Results

### 3.1. Sociodemographic Characteristics of the Participants

A total of 210 participants were involved in this study. That makes a 99.5% response rate. About 40% of the participants' ages were distributed between 17 and 19 years. The mean age of the participants was 18.7 years, with an SD of 3.9 years. More than one-half (122 or 58.1%) of the participants were single (Table 1).

### 3.2. Family History of the Participants

Table 2 describes the family history of the study participants. About 91 (43.3%) of the participants' families earn 1,000 ETB aid each month. Nearly one-half (103 or 49.0) of the participants' mothers were uneducated. About three-fourths of the participants were living with their families. About 150 (71.4%) of the participants perceived that they were not receiving enough money from their families.

### 3.3. Participants' Substance Use History

Regarding substance use, about 22 (10.5%) of the participants had ever smoked a cigarette. Of those, nearly three-fourths of the participants smoked occasionally. About 32 (15.2%) of the participants had ever drunk alcohol. Providentially, none of the participants used other illicit substances like hashish, cocaine, marijuana, or heroin other than cigarettes, alcohol, and khat (Table 3).

### 3.4. Prevalence of Rape

**Figure 1:**
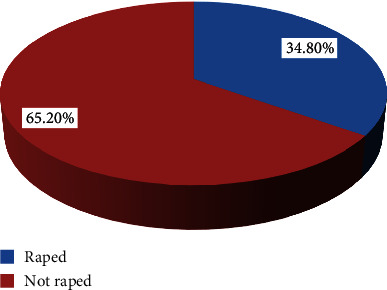
Prevalence of rape among female primary school students of Kule refugee camp, Gambella, southwest Ethiopia (*n* = 210).

**Figure 2:**
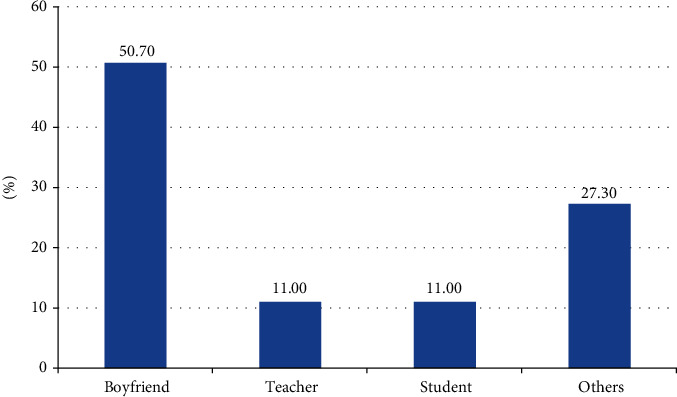
Perpetrators of rape among female primary school students of Kule refugee camp, Gambella, southwest Ethiopia (*n* = 73).

In this study, more than one-third (34.8%) of the participants were victims of rape. More than one-half (50.7%) were raped by their boyfriends (Figures 1 and 2).

### 3.5. Sexual History of the Participants

Table 4 displays the sexual histories of study participants. More than half of the participants had ever had sexual intercourse, and the mean age of their first sexual intercourse was 17.4 years (SD ± 2.2).

About 43 (34.4%) of the participants stated that peer pressure was the reason for their first sexual intercourse. Shockingly, the majority (79.5%) of those who experienced rape reported that their perpetrator did not use a condom. Around half of the participants mentioned that their schoolmates who confronted rape dropped out of school.

### 3.6. Factors Associated with the Prevalence of Rape

Table 5 describes both the bivariate and multivariate analysis outputs of factors associated with rape. In the bivariate analysis, variables such as cigarette smoking, alcohol consumption, khat chewing, having had a boyfriend, and the number of boyfriends showed an association with rape. However, in multivariate analysis, after controlling for potential confounders, smoking (AOR: 4.3; 95% CI: 1.61, 10.93), drinking alcohol (AOR: 3.2; 95% CI: 1.43, 7.03), and having a boyfriend (AOR: 2.81; 95% CI: 21, 4.05) were variables that showed an independent association with rape.

## 4. Discussion

The current study was aimed at assessing the prevalence of rape and its predictors among female students attending elementary schools in Kule refugee camp. Accordingly, it found 34.8% (95% CI: 28.1-41) of rape victims in the area. This finding is in line with a systematic review done by Araujo et al. [2]. It is also in line with a study conducted among refugees and asylum seekers in Belgium and the Netherlands, which reported a 33.4% rape prevalence [26].

Like in the WHO's and UNHCR's reports, substance use such as alcohol drinking and khat chewing was shown to have a significant association with being raped in the current study [7, 32]. The participants who drank alcohol were more than threefold more likely to be raped than those who did not drink. The likelihood of being raped was more than fourfold higher among participants who chewed tobacco than among those who did not. This finding is also consistent with a study conducted by Greathouse et al. [20]. The victim's unprotected behavior following drug usage might be the reason for this association.

Of the participants victimized by rape, 46.6% were raped in the community. This shows that the proportion of rape victims in the community was higher than that in school and at home. To the extent of our knowledge, there has been no previous study supporting the present finding, despite generally mentioning the social community as the place of rape and other sectarian violence. However, the reason could be insecurity and a lack of a supportive social environment in contrast to school and home.

The majority (79.5%) of the participants reported that they or the perpetrator did not use a condom. This finding was supported by Koss et al., who revealed that the majority of rape participants did not use condoms at the rape scene [10]. This could be due to the fact that the rapists have a ruthless approach and use forceful behavior that the victim cannot resist and has to negotiate to use condoms [24, 34, 35].

Around 50.7% of the participants were raped by their boyfriends. And also, having boyfriends was shown to have a significant association with suffering from rape. The odds of being raped were nearly three times higher among the participants who had had a boyfriend than among those who had not had a boyfriend. This finding is consistent with a study in Belgium and the Netherlands by Keygnaert et al. [26]. In addition, it was supported by the WHO's multicountry study [33]. This might be related to the potential influence of the boyfriend.

### 4.1. Strength and Limitations

As a strength, this study followed the Strengthening of Reporting of Observational Studies in Epidemiology (STROBE) checklist (S2 Table). However, this study utilized a small sample size, which could affect the power of the study. This study was also conducted on such a sensitive issue, in which some of the victims might not remember the scene they went through due to psychological pain or fear of stigma and shame. Therefore, these circumstances might understate the prevalence of rape in the study. Furthermore, this study's cross-sectional design constituted a weakness.

## 5. Conclusion

This study found a high prevalence of rape in the study area. The participants' behaviors, including having had a boyfriend, smoking, and drinking alcohol, were found to be factors exposing them to rape. Therefore, we recommend that the camp administration and humanitarian service organizations strengthen the preventive measures against rape and reinforce solid law enforcement against rapists.

## Figures and Tables

**Table 1:** Sociodemographic characteristics of female primary school students of Kule refugee camp, Gambella, southwest Ethiopia (*n* = 210).

Variable	Categories	Frequencies	Percentages
Age	≤13	12	5.7
	14-17	52	24.8
	17-19	87	41.4
	≥20	59	28.1

Marital status	Single	122	58.1
	Married	62	29.5
	Separated	24	11.4
	Others^a^	2	1.0

Religion	Adventist	60	28.6
	Muslim	2	1.0
	Protestant	99	47.1
	Catholic	37	17.6
	Others^b^	12	5.7

Ethnicity	Nuer	114	54.3
	Mundari	10	4.8
	Dinka	48	22.9
	Shilluk	38	18.1

Residence	Terkiedi	46	21.9
	Kule	132	62.9
	Nguenyiel	32	15.2

Grade	5^th^ and 6^th^	82	39.0
	7^th^ and 8^th^	128	61.0

^a^Divorce and widowed. ^b^Traditional believers and pagan.

**Table 2:** Family history of female primary school students of Kule refugee camp, Gambella, southwest Ethiopia (*n* = 210).

Variables	Categories	Frequency	Percentages
Family income (aid per family member in ETB)	500	65	31.0
	1000	91	43.3
	1200	54	25.7

Maternal educational status	Uneducated	103	49.0
	Elementary	52	24.8
	High school	35	16.7
	College and above	20	9.5

Paternal educational status	Uneducated	77	36.7
	Elementary	39	18.6
	High school	60	28.6
	College and above	34	16.2

Family living together	Yes	160	76.2
	No	50	23.8

Receiving enough from family	Yes	60	28.6
	No	150	71.4

Need support from family member	Yes	130	61.9
	No	80	38.1

Note: ETB: Ethiopian birr.

**Table 3:** Behavioral (substance use history) characteristics of female primary school students of Kule refugee camp, Gambella, southwest Ethiopia.

Variable	Categories	Frequency	Percentages
Smoking	Yes	22	10.5
	No	188	89.5
Frequency (*n* = 22)	Regularly	6	27.3
	Sometimes	16	72.7
Alcohol	Yes	32	15.2
	No	178	84.8
Frequency (*n* = 32)	Regularly	8	25.0
	Sometimes	24	75.0
Khat	Yes	12	5.7
	No	198	94.3
Frequency (*n* = 12)	Regularly	8	66.7
	Sometimes	4	33.3

**Table 4:** Sexual history of female primary school students of Kule refugee camp, Gambella, southwest Ethiopia.

Variables	Categories	Frequencies	Percentages
Having boyfriend or husband	Yes	129	61.4
	No	81	38.6

Number of boyfriend (*n* = 129)	One	85	65.9
	Two and above	44	34.1

Ever had sexual intercourse	Yes	125	59.5
	No	85	40.5

Reason for starting sexual intercourse (*n* = 125)	In marriage	52	41.6
	For financial purpose	10	8.0
	For passing exam	20	16.0
	By peer pressure	43	34.4

Age at first sexual intercourse (*n* = 125)	12-15	23	18.4
	16-19	85	68.0
	≥20	17	13.6

Place of forced sex (*n* = 73)	In home	18	24.7
	In school	12	16.4
	In community	34	46.6
	Others	9	12.3

Mechanism used to escape from the scene (*n* = 73)	By shouting	20	27.4
	By giving promise	26	35.6
	By fighting	14	19.2
	Others	13	17.8

Frequency of forced sex (*n* = 73)	One time	35	47.9
	Two times	21	28.8
	More than two times	17	23.3

Perpetrator used condom (*n* = 73)	Yes	15	20.5
	No	58	79.5

Know other girls who dropped out from school after experiencing rape (*n* = 210)	Yes	116	55.2
	No	94	44.8

**Table 5:** Bivariate and multivariate analyses of factors associated with rape among female primary school students of Kule refugee camp, Gambella, southwest Ethiopia.

Variables	Categories	Rape		COR (95% CI)	AOR (95% CI)
		Yes	No		
Smoking	Yes	14 (6.6%)	8 (3.8%)	3.8 (1.52, 9.62)	4.3 (1.61, 10.93)^∗^
	No	59 (28.1%)	129 (61.5%)	Ref	Ref
Drink alcohol	Yes	18 (8.6%)	14 (6.6%)	2.9 (1.34, 6.12)	3.2 (1.43, 7.03)^∗^
	No	55 (26.2%)	123 (58.6%)	Ref	Ref
Chewing khat	Yes	8 (3.8%)	4 (1.9%)	4.1 (1.19, 14.10)	3.9 (1.13, 13.92)
	No	65 (31.0%)	133 (63.3%)	Ref	Ref
Having boyfriend	Yes	53 (25.2%)	76 (36.2%)	2.1 (1.15, 3.93)	2.8 (1.21, 4.05)^∗∗^
	No	20 (9.6%)	61 (29.0%)	Ref	Ref
Number of boyfriend (*n* = 129)	One	22 (17.1%)	63 (48.8%)	0.13 (0.06, 0.30)	0.23 (0.10, 0.37)
	Two and above	32 (24.8%)	12 (9.3%)	Ref	Ref

^∗^ *p* value ≤ 0.05; ^∗∗^*p* value ≤ 0.001. AOR: adjusted odds ratio; CI: confidence interval; COR: crude odds ratio; Ref: reference group.

## Data Availability

All the datasets used in this study can be obtained from the corresponding author upon reasonable request.

## Supplementary Materials

Supplementary 1 S1 File: information sheet and consent form used to assess the prevalence of rape and its predictors among female students attending elementary schools: in the case of Kule refugee camp, Gambella, southwest Ethiopia.

Supplementary 2 S1 Table: questionnaire used to assess the prevalence of rape and its predictors among female students attending elementary schools: in the case of Kule refugee camp, Gambella, southwest Ethiopia.

Supplementary 3 S2 Table: Strengthening of Reporting of Observational Studies in Epidemiology (STROBE) statement checklist used to assess the prevalence of rape and its predictors among female students attending elementary schools: in the case of Kule refugee camp, Gambella, southwest Ethiopia.
