# Supplementary material for: Prevalence of Rape and Its Predictors among Female Students Attending Elementary Schools: In the Case of Kule Refugee Camp, Gambella, Southwest Ethiopia—A Cross-Sectional Study
Source: Int J Reprod Med. 2023 Jun 2;2023:5559246. doi: 10.1155/2023/5559246 (PMC10256440; doi:10.1155/2023/5559246)
Supplement: Supplementary 1 — S1 File: information sheet and consent form used to assess the prevalence of rape and its predictors among female students attending elementary schools: in the case of Kule refugee camp, Gambella, southwest Ethiopia. [file 5559246.f1.docx]

**S1 File: Information sheet and consent form used to assess the prevalence of rape and its predictors among female students attending elementary schools: in case of Kule refugee camp, Gambella, southwest Ethiopia**

Greetings, this is Bhan Sudan Kong. I am a 4^th^-year nursing graduating class student at Mettu University.

I’m conducting the survey in Kule refugee camps, Gambella, southwest Ethiopia, among elementary school female students with the research entitled ***"Prevalence of rape and its predictors among female students attending elementary schools in Kule Refugee Camp, Gambella, Southwest Ethiopia, 2022*".** You are selected to participate in this study. The findings of the study will be used for better planning and intervention of rape against females attending elementary schools. Based on survey results, possible suggestions will be given to refugee and school administrators and program planners to provide the most efficient and continuous flow of on-rape information.

The questionnaire takes only 15–30 minutes to fill out. Your suspense will be made anonymous so that you can freely express your opinion and fill out the choice, but discussion with friends is not allowed. Remember, your name is not recorded and no one will be able to find out who filled out this questionnaire. If you need clarification, you can ask the facilitator. Participation will be voluntary and participants may withdraw from the study at any stage or time without explanation, penalty, or loss of benefit. Please give only what you are asked to answer for each question by encircling the letter and filling in the blank space.

Are you willing to participate in this study?  Yes _________           No________

**Thank you for your genuine cooperation!!**
