# Supplementary material for: Prevalence of Rape and Its Predictors among Female Students Attending Elementary Schools: In the Case of Kule Refugee Camp, Gambella, Southwest Ethiopia—A Cross-Sectional Study
Source: Int J Reprod Med. 2023 Jun 2;2023:5559246. doi: 10.1155/2023/5559246 (PMC10256440; doi:10.1155/2023/5559246)
Supplement: Supplementary 2 — S1 Table: questionnaire used to assess the prevalence of rape and its predictors among female students attending elementary schools: in the case of Kule refugee camp, Gambella, southwest Ethiopia. [file 5559246.f2.docx]

**S1 Table: Questionnaire used to assess prevalence of rape and its predictors among female students attending elementary schools: in case of Kule refugee camp, Gambella, southwest Ethiopia**

**Part one: Socio demographic characteristic other related questions**

| 1.1 | How old are you? |  |
| --- | --- | --- |
| 1.2 | What is your religion? | A/ Adventist  B/Muslim  C/ Protestant  D/Catholic  E/Other |
| 1.3 | What is your marital status? | A/Single  B/married  C/living with partner  D/other |
| 1.4 | What is your ethnicity | A/Nuer  B/Oromo  C/ Dinka  D/ Shilluk  E/others |
| 1.5. | Residence | A/ Terkiedi camp  B/ Kule camp  C/ Nguenyiel camp |
| 1.6. | Class | A/ 5^th^  B/ 6^th^  C/ others |
| 1.7. | How do you perceive the income status of your family? | A/500  B/1000  C/>1000 |

**Part two: family history**

| 2.1. | 2.1. Are your father and mother living together currently? | A/yes  B/no |
| --- | --- | --- |
| 2.2. | Family educational status | A/illiterate  B/ Elementary  C /High school  D/ college and above |
| 2.3. | Do you think that you are receiving enough money according to your demand? | A/Yes  B/ No  C/Poor |
| 2.4. | When you need help or problems can you usually count on family members for support? | A/ Yes  B/ No |

**Part three: Individual behavioral factors**

| 3.1. | Do you smoke? | A/Yes  B/No |
| --- | --- | --- |
| 3.2. | If yes(Q 3.1) How often do you smoke? | A/Regularly  B/Sometimes |
| 3.3. | Do you drink alcohol | A/ yes B/ no |
| 3.4. | If so how often do you drink? | A/Regularly  B/Some times |
| 3.5. | Do you chew khat**?** | A/ Yes  B/ NO |
| 3.6. | If yes how often do you chew? | A/ Sometimes  B/regularly |

| **Part Four. sexual history**   \| 4.1 \| Do you have regular boyfriend currently? \| A/yes  B/No \| \| --- \| --- \| --- \| \| 4.2 \| Have you ever had sexual intercourse? \| A/yes  B\ no \| \| 4.3. \| If yes for (Q 4.2) how do you start sexual intercourse? \| A /In a marriage  B/ For financial purpose  C/ For passing examination D/ Peer pressure \| \| 4.4. \| What was your age at first sexual intercourse? \| In Years………………… \| \| 4.5. \| Have You encountered forced sex? \| A/ Yes  B/ No \| \| 4.6 \| If yes(Q 4.5) where was the place? \| A/ In my home  B/ In school  C /In the community  D /Other \| \| 4.7. \| If encountered forced sex in your life time who was the perpetuator? \| A/ Boyfriend  B/ Teacher  C /Student D /Other \| \| 4.8. \| If you have ever been forced into unwanted sexual Intercourse and escaped. How was mechanism of Escape? \| A/ By shouting/crying  B /By giving promise  C /By Fighting  D Other \| \| 4.9. \| How many times in your life time do you encountered Such forced sex? \| A/ One times  B/ Two times  C/ more than two times \| \| 4.10. \| How many sexual partners or boyfriends have you Experiences until now? \| A/ nothing  B /One times  C/ more than one times \| \| 4.11 \| If your answer for Q (4.9) is more than one did you/your Partner use condom? \| A/ Yes B /No \| \| 4.12 \| Do you know other girls who dropped out from school after experienced complete rape? \| A/yes B/no \|     **Thank you for taking your time to give answer!!!!** |
| --- | --- | --- | --- | --- | --- | --- | --- | --- | --- | --- | --- | --- | --- | --- | --- | --- | --- | --- | --- | --- | --- | --- | --- | --- | --- | --- | --- | --- | --- | --- | --- | --- | --- | --- | --- | --- |
